# Gastrointestinal hemorrhage before anticoagulant therapy in Kawasaki disease: a case report

**DOI:** 10.1186/s12887-020-1916-6

**Published:** 2020-01-27

**Authors:** Chenmin Hu, Yanping Yu

**Affiliations:** Department of Pediatrics, Affiliated Hangzhou First People’s Hospital, Zhejiang University School of Medicine, Hangzhou, 310006 China

**Keywords:** Kawasaki disease, Gastrointestinal hemorrhage, Duodenal ulcer, Abdominal pain, Case report

## Abstract

**Background:**

Kawasaki disease (KD) is an acute febrile multisystem vasculitis and has been recognized to be the most common cause of acquired heart disease in children. Owing to its propensity to involve vessels throughout the entire body, KD often mimics other disease processes. The diagnosis might be delayed if other prominent symptoms appear before the characteristic clinical features of KD. Although gastrointestinal symptoms including vomiting, diarrhea, and abdominal pain are not uncommon in KD patients, KD with gastrointestinal bleeding is quite rare.

**Case presentation:**

A previously healthy 4-year-old boy initially presented with abdominal pain, followed by fever, rash, and gastrointestinal hemorrhage, eventually diagnosed as complete KD. The patient recovered smoothly after appropriate management and no subsequent complications occurred in the following months.

**Conclusion:**

The diagnosis of KD should be considered in children presenting with abdominal symptoms and fever without definable cause. Pediatricians should be aware of the risk of gastrointestinal bleeding in patients with KD, especially in those with prominent abdominal symptoms.

## Background

Kawasaki disease (KD) is an acute febrile multisystem vasculitis of unknown etiology, especially involving coronary arteries, and has been recognized to be the most common cause of acquired heart disease in children [1]. This vasculitis results in exudative change with infiltration of lymphocytes and large mononuclear cells into the media and adventitia of the vessels with endothelial necrosis [1]. Although inflammation of the coronary arteries results in the most important clinical outcomes, these problems of vessels may also occur in many other organs. Although the prevalence of gastrointestinal involvement in KD is unknown, gastrointestinal symptoms including vomiting, diarrhea, and abdominal pain are not uncommon [2]. However, gastrointestinal hemorrhage in patients with KD is rather rare. Herein, we report a 4-year-old boy with KD initially presenting with abdominal pain and complicated by gastrointestinal hemorrhage due to duodenal ulcer before the use of aspirin. Favorable outcomes for these patients may depend upon the keen judgment of pediatricians familiar with the disease’s presentation and management.

## Case presentation

A previously healthy 4-year-old boy presented with a 5-day history of abdominal pain, 4-day history of fever and rash. Five days ago, the boy had a sudden onset of persistent abdominal pain, accompanied by vomiting. Empirically, initial therapy with intravenous antibiotics and cimetidine was performed in local hospital. The next day, fever occurred (39 °C–39.8 °C), accompanied by a miliary rash scattered throughout the body. On the third day, the skin rash increased, accompanied by conjunctival hyperemia, erythema and swelling of the hands and feet. The laboratory examinations revealed white blood cell count of 13.14 × 10^9/L with predominance of neutrophil 90.6%, hemoglobin level of 120 g/L, platelet count of 204 × 10^9/L, and C-reactive protein (CRP) of 101.9 mg/L. Abdominal computed tomography shows the accumulation of gas and fluid in the small intestine. An echocardiography showed no dilatation of coronary arteries. On day 5, He vomited a small amount of coffee-like liquid and discharged a moderate amount of tarry stool. Then, the patient was transferred to our hospital by emergency.

On admission, physical examination revealed an acutely ill boy with fever of 38.5 °C, pulse of 124 times per minute, breathing of 30 times per minute, and blood pressure of 94/47 mmHg. The boy displayed erythematous maculopapular rash over the body, bilateral conjunctival hyperemia, erythema and swelling of the hands and feet, dry and cracked lips, oropharyngeal hyperemia and mild strawberry tongue. One enlarged lymph node approximately 1.5 × 1 cm was palpable in the right anterior cervical triangle. His abdomen was slightly tense with mild tenderness. Laboratory tests are summarized in Table 1. The main abnormalities were as follows: hemoglobin concentration 108 g/L; white blood cell count 11.5 × 10^9/L, neutrophils 91.5%; platelet count 65 × 10^9/L; CRP 31 mg/L, serum alanine aminotransferase (ALT) 54 U/L, total protein 37.2 g/L, albumin 21.3 g/L, creatine kinase isoenzyme MB (CK-MB) 56 U/L, potassium 3.11 mmol/L, D-Dimer 6230 μg/L, fecal occult blood +++. In light of these findings and the clinical features, a diagnosis of KD with upper gastrointestinal bleeding was made. The boy received supportive care and intravenous omeprazole (0.6 mg/kg/day). On day 6 (the 5th day of fever), a total dose of intravenous immunoglobulin (IVIG, 2 g/kg) was administered. Aspirin was not administered because of his upper gastrointestinal bleeding. After 24 h of IVIG, his fever began to subside and the skin rash diminished dramatically. On the second day after IVIG, laboratory findings showed a normal leukocytes of 6.7 × 10^9/L and a remarkable fall of the CRP levels at 5 mg/L and the D-Dimer levels at 1280 μg/L (Table 1). However, the fecal occult blood continued to be positive although his gastrointestinal symptoms were improved.

**Table 1:** Laboratory data during acute phase

Parameters	Day 3	Day 5	Day 8	Day 12
Leukocytes (× 10^9/L)	13.14	11.5	6.7	9.8
Neutrophil (%)	90.6	91.5	40.1	57.0
Hemoglobin (g/L)	120	108	102	103
Platelet (×10^9/L)	204	65	115	672
C-reactive protein (mg/L)	101.9	31	5	3
Total protein (g/L)	/	37.2	51	52.3
Albumin (g/L)	/	21.3	21.7	25.9
Alanine aminotransferase (U/L)	/	54	19	11
Total bilirubin (μmol/L)	/	6.3	6.6	5.2
Creatinine (μmol/L)	/	59	47	37
Creatine kinase (U/L)	/	61	39	33
Creatine kinase isoenzyme MB (U/L)	/	56	27	23
Potassium (mmol/L)	/	3.11	3.59	4.47
Sodium (mmol/L)	/	131	132	135
Chlorine (mmol/L)	/	99	101	105
Prothrombin time (sec)	/	13.3	12.5	/
D-Dimer (μg/L)	/	6230.0	1280	700.0
Serum amylase (U/L)	/	33	/	/
Fecal occult blood	/	+++	+	/

**Fig. 1:**
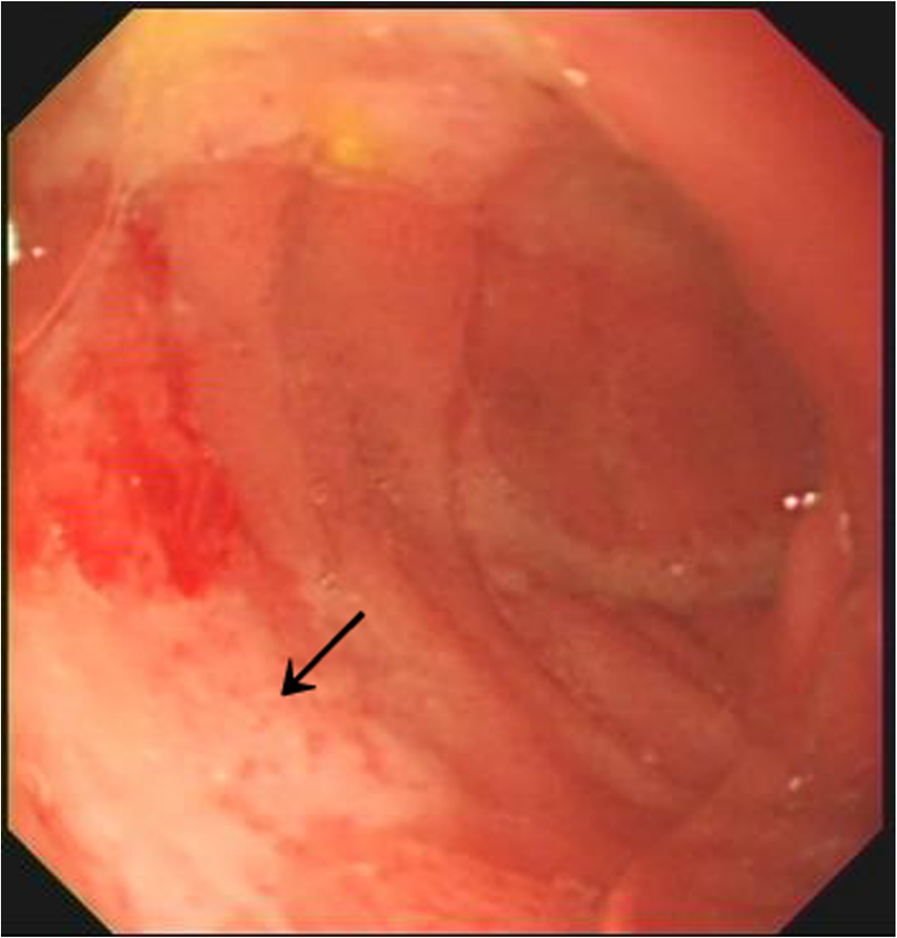
Gastroscopy results showing an active ulcer covered with thick “white moss” in duodenal bulb

An endoscopy was performed on Day 8 of illness. We found an active ulcer covered with thick “white moss” in duodenal bulb (Fig. 1). Then, the dose of intravenous omeprazole was increased to 0.8 mg/kg/day. Repeated echocardiography on Day 9 demonstrated a mild dilatation of the left coronary artery (2.8 mm). Oral clopidogrel (1 mg/kg/day) was added to treatment. On day 10, 13C-urea breath test showed that his helicobacter pylori status was negative. On day 12, most of the laboratory parameters were normal or close to normal except for elevated platelet count of 672 × 10^9/L. The next day, he was discharged and continued with oral omeprazole (0.6 mg/kg/day) for 4 weeks and clopidogrel (1 mg/kg/day) for a further 8 weeks. Follow-up at 1 month after discharge revealed no recurrence of fever or remarkable abdominal pain. Repeated echocardiography at 8 weeks after discharge revealed that the dilated left coronary artery regressed to normal. A 1 year follow-up showed no cardiac involvement or other complications.

## Discussion

KD is the most common vasculitis in children [3]. Even though more than 50 years have passed since the first description of this disease, the diagnosis of KD continues to remain a clinical dilemma and there is no confirmatory laboratory test [4]. The diagnosis of KD is based on the presence of fever (lasting > 5 days) together with 4 of the 5 characteristic diagnostic criteria (rash, oropharyngeal changes, non-purulent conjunctivitis, cervical lymphadenopathy and change of the extremities) [1, 4]. A prompt diagnosis is crucial because prognosis relies on timely initiation of the optimal treatment. The most common and life-threatening complication of KD, coronary artery abnormalities (CAA), will occur in approximately 25% of untreated patients which may lead to death by causing coronary thrombosis or myocardial infarction. The delayed diagnosis of KD could result in life-threatening problems particularly including cardiovascular sequelae.

Owing to its propensity to involve the medium-sized arteries in multiple organs, KD often mimics other disease processes. The diagnosis might be delayed if other prominent symptoms appear before the characteristic clinical features of KD, especially in children with incomplete or atypical forms. Incomplete KD occurs in patients presenting a typical fever without a sufficient number of main clinical criteria, with or without CAA. This kind of KD is frequent in children younger than 12–24 months and should be considered in the differential diagnosis of prolonged unexplained fever in childhood associated with any of the characteristic clinical features of the disease [1]. The diagnosis can be considered confirmed when coronary artery aneurysms are identified in such patients by echocardiography. However, coronary artery dilatation is generally not detected by echocardiography until after the first week of illness, and a normal echocardiogram in the first week of illness does not rule out the diagnosis of KD. The term atypical KD is used when there are one or more symptoms or signs different from the main KD clinical features such as pneumonia, meningoencephalitis, facial paralysis, acute abdomen, arthritis, cholestatic jaundice, and renal injury [5]. The diagnosis of KD is more challenging in the presence of one or more atypical manifestations.

The prevalence of gastrointestinal involvement in KD is unknown as available data can only be derived from single case and case series. Zulian et al. in their case series reported an incidence of atypical KD with a clinical onset characterized by acute abdomen of 4.6% [6]. Gastrointestinal symptoms at KD onset can complicate clinical recognition, lead to unnecessary invasive interventions and cause therapeutic delay, thus increasing the risk of cardiac complications [2]. A retrospective multicenter report from Italy demonstrated that presenting gastrointestinal symptoms in KD identify patients at higher risk for IVIG-resistance and for the development of coronary aneurysms [7]. Therefore, the diagnosis of KD should be considered in children with gastrointestinal symptoms and prolonged fever without clear cause.

Our patient presented with abdominal pain at the onset of disease. Fortunately, the followed fever, rash and other typical signs of KD led to timely diagnosis and treatment. Although laboratory tests are nonspecific, they can provide support for a diagnosis of KD in patients with suggestive clinical features. For our patients, laboratory tests supporting KD diagnosis included changes of markers at acute phase such as leukocytosis, elevated CRP, hypoalbuminemia, mild elevations of ALT, and mild elevations of CK-MB and thrombocytosis on day 12 of illness. Thrombocytosis is a characteristic feature of KD but generally does not occur until the second week, peaking in the third week and normalizing by 4 to 6 weeks after onset in most cases [1, 5]. In addition, thrombocytopenia and elevations of D-Dimer in our patient were detected in the first week of illness. Although uncommon, thrombocytopenia in acute phase of KD may be a sign of disseminated intravascular coagulation and is a risk factor for the development of coronary artery abnormalities.

The mechanism of gastrointestinal involvement at the onset of KD has not yet been clarified. The possible explanation is that gastrointestinal tract is the first organ involved with systemic vasculitis of the medium-sized arteries, leading to abdominal pain and vomiting. However, the etiology and the entire pathophysiology of KD remain elusive, despite a host of data supports the notion that the pathogenesis of KD is closely associated with dysregulation of immune responses to infectious agents [8, 9]. The gastrointestinal tract has been proposed as a major site of entry of infectious agents that might act as superantigens, even though more recent studies have favored a canonical response to a conventional antigen [1]. Recently, an increasing number of studies have focused on the contribution of gut microbiota to KD. Esposito et al. postulated that an imbalance in the gut microbiota might indirectly interfere with the normal function of immunity, and that variable microbiota interactions with environmental factors, mainly infectious agents, might selectively drive the development of KD in genetically susceptible children [10].

Compared to the frequently reported gastrointestinal symptoms, KD with gastrointestinal hemorrhage is quite rare. Only 5 previous cases have been documented in the English literature, as showed in Table 2. Two cases of severe gastrointestinal hemorrhage complicating high-dose aspirin therapy were iatrogenic [11]. Zulian et al. reported a 20-month-old KD boy who had sudden onset of hematemesis without aspirin and presented with diffuse hemorrhagic duodenitis by endoscopy [6]. Singh et al. reported a 4.5-year-old KD boy who developed hemorrhagic shock and revealed a duodenal ulcer with active bleeding by surgery [12]. Chang et al. reported a 5-year-old KD boy who had tarry stool on day 6 of fever. Three days later, he was still treated with high-dose aspirin and massive gastrointestinal bleeding occurred on day 5 of aspirin treatment [13].

**Table 2:** Clinical characteristics of the reported patients with gastrointestinal hemorrhage in Kawasaki disease

Year	First author	Age/sex	Clinical onset	Details of gastrointestinal bleeding	Blood transfusion	Endoscopy	Surgery
1996	Matsubara	2 y/M	Fever	Hematemesis on day 19 of illness (day 13 of aspirin)	Yes	Revealed a 2 cm duodenal ulcer	None
1996	Matsubara	4 y/F	Fever	Melanotic stools followed by emesis of blood on day 31 of illness (day 26 of aspirin)	Yes	None	None
2003	Zulian	20 m/M	Fever	Hematemesis on day 7 of illness without aspirin	Yes	Showed diffuse hemorrhagic duodenitis	None
2004	Chang	5 y/M	Fever, cervical lymphadenitis	Tarry stool on day 6 of illness; Massive gastrointestinal bleeding on day 14 of illness (day 5 of aspirin)	Yes	NA	NA
2007	Singh	4.5 y/M	Nausea, vomiting, diarrhea and fever	Hemorrhagic shock with hematemesis and hematochezia at 2 months after onset (without aspirin)	Yes	Failed because of the massive bleeding	Revaeled a 1.5 cm duodenal ulcer with active bleeding

*M* male, *F* female, *y* year, *m* month, *NA* not available

Before the definitive diagnosis of KD, our patient developed upper gastrointestinal bleeding confirmed by the subsequent endoscopy. On day 5 of fever, he received intravenous immunoglobulin and omeprazole but not high-dose aspirin. The iatrogenic second bleeding as in Chang’s case was avoided. Generally, when high-dose aspirin is discontinued after KD patient has been afebrile for 48 to 72 h, low-dose aspirin (3–5 mg/kg/d) should be started and continued until the patient has no evidence of coronary changes by 6 to 8 weeks after onset of illness. For children who develop coronary abnormalities, aspirin may be continued indefinitely. Clopidogrel (0.2–1 mg/kg/d) can be used in place of low-dose aspirin for patients who are aspirin resistant or allergic to aspirin [1]. For our patient, aspirin may have a higher risk of aggravating duodenal ulcer. As an alternative anticoagulant therapy, clopidogrel was started and continued for 8 weeks after discharge when a mild dilatation of coronary artery was revealed on day 9 of illness. Finally, our patient completely recovered.

IVIG and aspirin were considered as the standard initial treatment of KD for decades. The efficacy of IVIG administered in the acute phase of KD is well established to reduce the prevalence of CAA [1]. However, the role of high-dose aspirin in KD remains controversial. Kuo et al. demonstrated that high-dose aspirin in KD did not confer any benefit with regards to inflammation and it did not appear to improve treatment outcomes [14]. A recent meta-analysis revealed that low-dose aspirin (3–5 mg/kg/d) may be as effective as the use of high-dose aspirin (> 30 mg/kg/d) for the initial treatment of KD [15]. Accordingly, we believe that high-dose aspirin may be unnecessary in KD with prominent gastrointestinal symptoms. However, further well-designed prospective studies are still required to evaluate the efficacy of low-dose aspirin for KD.

## Conclusions

The diagnosis of KD should be considered in children presenting with abdominal symptoms and fever without definable cause. Pediatricians should be aware of the risk of gastrointestinal bleeding in patients with KD, especially in those with prominent abdominal symptoms.

## Data Availability

The datasets used and/or analyzed during the current study are available from the corresponding author on reasonable request.

## Abbreviations

ALT: Alanine aminotransferase
CAA: Coronary artery abnormalities
CK-MB: Creatine kinase isoenzyme MB
CRP: C-reactive protein
IVIG: Intravenous immunoglobulin
KD: Kawasaki disease

## References

[1] McCrindle BW, Rowley AH, Newburger JW (2017). Diagnosis, treatment, and long-term Management of Kawasaki Disease: a scientific statement for health professionals from the American Heart Association. Circulation.

[2] Colomba C, La Placa S, Saporito L (2018). Intestinal Involvement in Kawasaki Disease. J Pediatr.

[3] Singh S, Vignesh P, Burgner D (2015). The epidemiology of Kawasaki disease: a global update. Arch Dis Child.

[4] Singh S, Jindal AK, Pilania RK (2018). Diagnosis of Kawasaki disease. Int J Rheum Dis.

[5] Marchesi A, Tarissi de Jacobis I, Rigante D (2018). Kawasaki disease: guidelines of the Italian Society of Pediatrics, part I - definition, epidemiology, etiopathogenesis, clinical expression and management of the acute phase. Ital J Pediatr.

[6] Zulian F, Falcini F, Zancan L (2003). Acute surgical abdomen as presenting manifestation of Kawasaki disease. J Pediatr.

[7] Fabi M, Corinaldesi E, Pierantoni L (2018). Gastrointestinal presentation of Kawasaki disease: a red flag for severe disease?. PLoS One.

[8] Principi N, Rigante D, Esposito S (2013). The role of infection in Kawasaki syndrome. J Inf Secur.

[9] Nakamura A, Ikeda K, Hamaoka K (2019). Aetiological significance of infectious stimuli in Kawasaki disease. Front Pediatr.

[10] Esposito S, Polinori I, Rigante D (2019). The gut microbiota-host partnership as a potential driver of Kawasaki syndrome. Front Pediatr.

[11] Matsubara T, Mason W, Kashani IA, Kligerman M, Burns JC (1996). Gastrointestinal hemorrhage complicating aspirin therapy in acute Kawasaki disease. J Pediatr.

[12] Singh R, Ward C, Walton M (2007). Atypical Kawasaki disease and gastrointestinal manifestations. Paediatr Child Health.

[13] Chang CH, Chen MH, Yang W (2004). Kawasaki disease presenting with lymphadenopathy and gastrointestinal hemorrhage: report of one case. Acta Paediatr Taiwan.

[14] Kuo HC, Lo MH, Hsieh KS, Guo MM, Huang YH (2015). High-Dose Aspirin is Associated with Anemia and Does Not Confer Benefit to Disease Outcomes inKawasaki Disease. PLoS One.

[15] Zheng X, Yue P, Liu L (2019). Efficacy between low and high dose aspirin for the initial treatment of Kawasaki disease: Current evidence based on a meta-analysis. PLoS One.

